# Impact of smoking on gremlin-1, syndecan-4, and IL-1β responses to non-surgical periodontal therapy in stage 3 periodontitis: a comparative study with periodontally healthy controls

**DOI:** 10.1007/s00784-026-06859-y

**Published:** 2026-04-11

**Authors:** Yunus Emre Bayrakdar, Tuğba Aydın, Esra Laloğlu

**Affiliations:** 1 Department of Periodontology, Faculty of Dentistry, Atatürk University, Erzurum, 25000 Türkiye; 2 Department of Biochemistry, Faculty of Medicine, Atatürk University, Erzurum, Türkiye

**Keywords:** Gremlin-1, IL-1β, NSPT, Syndecan-4, Periodontitis

## Abstract

**Objectives:**

This study aimed to compare the periodontal status of periodontally healthy individuals with those of smokers and non-smokers with stage 3 periodontitis before and after treatment, as well as to investigate the levels of Gremlin-1, Syndecan-4, and IL-1β in GCF samples. Another objective of the study was to evaluate the relationship between these biomarkers and periodontitis and to assess the effects of smoking on their levels.

**Materials and methods:**

A total of 45 volunteers, 15 smokers (Group SP) and 15 non-smokers (Group NP) diagnosed with stage 3 periodontitis between the ages of 18 and 60, and 15 healthy individuals (Group H) were included in the study.

**Results:**

All clinical parameters of the SP and NP groups were found to be significantly higher than those of the H group before NSPT (*p*<0.001), and a significant reduction was observed in all clinical parameters after phase I treatment (*p*<0.001). Following NSPT, significant improvement was observed in all periodontal parameters in both periodontitis groups (*p*<0.001). Gremlin-1 levels in the SP and NP groups were statistically significantly lower after treatment compared to pre-treatment levels (p<0.05). While there was no statistically significant difference in IL-1β levels before and after treatment in the SP group (*p*>0.05), a statistically significant reduction was observed in the NP group after treatment (*p*<0.05). Similarly, no statistically significant difference was found in Syndecan-4 levels before and after treatment in the SP group (*p*>0.05), whereas the post-treatment measurements of the NP group were statistically significantly lower (*p*<0.05).

**Conclusions:**

After NSPT, Gremlin-1 levels in GCF decreased in both groups, whereas significant reductions in Syndecan-4 and IL-1β levels were observed only in non-smokers. These findings suggest that the regulatory effect of smoking on biomarkers may negatively influence the periodontal treatment response.

**Clinical relevance:**

Smoking is a major risk factor for periodontitis and may impair healing following nonsurgical periodontal therapy. While similar clinical improvements were observed, reductions in IL-1β and Syndecan-4 occurred only in non-smokers, whereas Gremlin-1 decreased in both groups. These findings suggest that smoking may influence molecular healing responses, and biomarker monitoring may support personalized periodontal management.

## Introduction

Periodontitis is a multifactorial inflammatory disease associated with dental plaque accumulation and characterized by complex interactions between pathogenic microorganisms, host immune responses, and environmental risk factors such as smoking [1]. Smoking alters both the microbial composition of dental plaque and the host immune response, while also reducing gingival blood flow, thereby accelerating periodontal tissue destruction [2]. Regular consumption of 10 or more cigarettes per day significantly increases the rate of disease progression and contributes to more advanced stages of periodontitis [3]. However, current diagnostic approaches remain insufficient for accurately reflecting disease activity and predicting future progression, highlighting the need for reliable biological markers to support early diagnosis and therapeutic decision-making.

Recent studies have suggested that Gremlin-1 and syndecans may serve as potential biomarkers in periodontal disease. Gremlin-1, a member of the transforming growth factor-β (TGF-β) superfamily, functions as an antagonist of bone morphogenetic proteins (BMPs) and has been implicated in inflammatory and tissue remodeling processes [4]. It has been shown to promote inflammatory disease progression through endothelial cell activation [5] and regulation of the nuclear factor-kappa B (NF-κB) signaling pathway [6]. Moreover, Gremlin-1 has been detected in periodontal tissues, where it appears to exert site-specific effects on cell proliferation and differentiation [7].

Syndecans are transmembrane heparan sulfate proteoglycans involved in cell signaling, inflammation, and tissue repair. Through interactions between the extracellular matrix and intracellular signaling pathways, syndecans regulate growth and differentiation in multiple tissues [8–10]. Syndecan-4, in particular, is expressed by immune cells within inflamed periodontal tissues and participates in diverse biological processes including tumorigenesis and inflammatory regulation [11–14]. Although increased Syndecan-4 expression has been observed in experimental periodontitis models, its precise role in human periodontal disease remains unclear [15].

Interleukin-1β (IL-1β) is a key proinflammatory cytokine that plays a central role in immune regulation and periodontal tissue destruction. Elevated IL-1β levels in gingival crevicular fluid (GCF) have been consistently associated with active periodontal inflammation and tissue breakdown [16, 17]. Nonsurgical periodontal therapy (NSPT) remains the cornerstone of periodontal infection control; however, limited data are available regarding the combined effects of NSPT on Gremlin-1, Syndecan-4, and IL-1β levels in smokers and non-smokers with Stage III periodontitis. Therefore, the present clinical and biochemical study aimed to investigate the impact of NSPT on these biomarkers in periodontally healthy individuals and patients with Stage III periodontitis, with particular emphasis on the modulatory effects of smoking.

## Materials & methods

The study protocol was approved by the Erzurum University Faculty of Medicine Clinical Research Ethics Committee (January 10, 2024; approval no. 01/16) and conducted in accordance with the Declaration of Helsinki (1975, revised 2013). The clinical trial was registered at ClinicalTrials.gov, a primary registry of the World Health Organization International Clinical Trials Registry Platform (registration number: NCT06687005). Written informed consent was obtained from all participants prior to enrollment.

A power analysis was performed to determine the required sample size to detect a 60% reduction in IL-1β levels, based on data from a previous study comparing smokers and non-smokers with periodontitis [18]. An effect size of 1.4 was calculated, and analysis was conducted using G*Power version 3.1 (Heinrich-Heine University Düsseldorf, Germany) with 95% power, a significance level of 0.05, and a two-tailed test. The results indicated that a minimum of 15 participants per group was required.

**Fig. 1:**
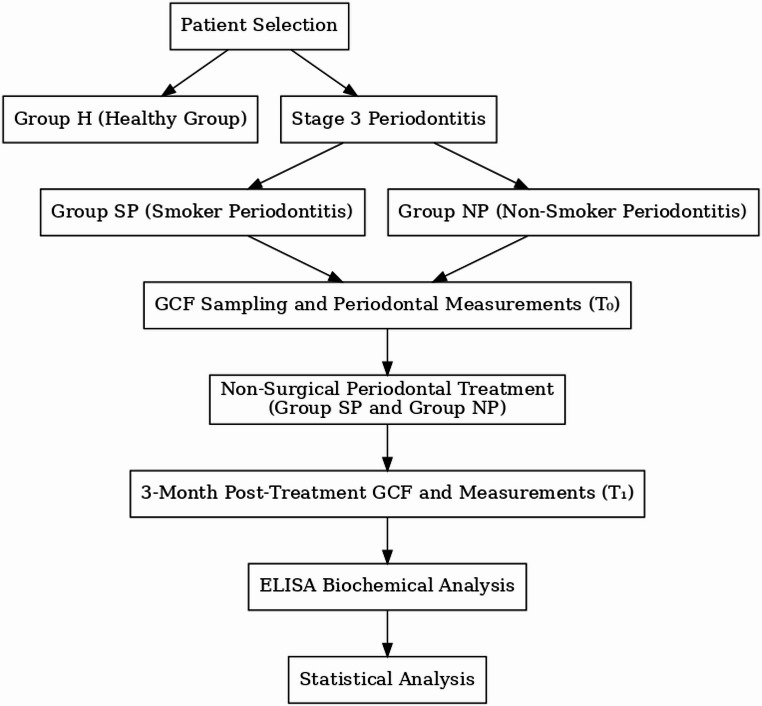
Study flow diagram

A total of 45 individuals attending the Periodontology Clinic of Atatürk University Faculty of Dentistry were recruited and classified according to the 2017 World Workshop on the Classification of Periodontal and Peri-Implant Diseases. Participants were categorized into three groups based on periodontal status and smoking history: periodontally healthy controls (Group H, *n* = 15), non-smokers with Stage III periodontitis (Group NP, *n* = 15), and smokers with Stage III periodontitis (Group SP, *n* = 15). The study flow is illustrated in Fig. 1.

Clinical periodontal measurements and gingival crevicular fluid (GCF) samples were obtained at two time points: baseline before treatment (T0) and three months after nonsurgical periodontal therapy (T1).

Smoking status was determined based on self-reported smoking history. Individuals who reported smoking more than 10 cigarettes per day were classified as smokers and included in the smoker periodontitis (SP) group. Smoking status was assessed at baseline through structured clinical interviews. Participants in the non-smoker periodontitis (NP) group and the periodontally healthy control (H) group reported no current or past smoking history.

### Inclusion criteria

Group H: Periodontally healthy individuals with no clinical signs of periodontal disease and no history of smoking.

Group NP: Non-smokers diagnosed with Stage III periodontitis, defined by probing pocket depth (PPD) ≥ 5 mm and clinical attachment level (CAL) ≥ 4 mm affecting ≥ 30% of teeth, with radiographic evidence of alveolar bone loss.

Group SP: Current smokers diagnosed with Stage III periodontitis based on the same clinical and radiographic criteria, with a smoking history of at least 10 cigarettes per day for a minimum of 3 years.

### Exclusion criteria

Participants were excluded if they were pregnant or breastfeeding, using oral contraceptives, had systemic diseases, had received periodontal therapy or antibiotic treatment within the previous 6 months, had used anti-inflammatory medication within the past 3 months, or declined participation.

### Clinical examination

Medical and dental histories were recorded for all participants, followed by comprehensive clinical and radiographic examinations. Smoking status was determined by self-report. Clinical measurements were performed by a single calibrated examiner (Y.E.B.) using a manual periodontal probe.

The evaluated periodontal parameters included Plaque Index (PI), Gingival Index (GI), Bleeding on Probing (BOP), Probing Pocket Depth (PPD), and Clinical Attachment Level (CAL). Measurements were obtained at six sites per tooth and rounded to the nearest 0.5 mm. To assess intra-examiner reliability, duplicate measurements were performed two hours apart.

### Gingival crevicular fluid collection

GCF samples were collected 24 h after clinical examinations at each time point to avoid interference with fluid flow. After removal of supragingival plaque and isolation with cotton rolls, standardized paper strips (Periopaper™, Oraflow Inc., Plainview, NY, USA) were gently inserted into the gingival sulcus for 30 s. Strips contaminated with blood or saliva were discarded. Samples were placed in Eppendorf tubes containing 500 µL phosphate-buffered saline and stored at − 80 °C until ELISA analysis.

### Biomarker analysis

Gremlin-1, IL-1β, and Syndecan-4 levels were measured by commercially available ELISA kits (BT LAB, China) according to manufacturer protocols. Samples were analyzed in duplicate by a laboratory investigator blinded to group allocation.

### Nonsurgical periodontal therapy

All participants in the periodontitis groups received full-mouth scaling and root planing, along with saline irrigation. Subgingival instrumentation was performed using Gracey curettes until smooth root surfaces were achieved. Oral hygiene instructions were provided, including the use of interdental brushes and dental floss. No chemical plaque control agents were prescribed.

### Statistical analysis

Statistical analyses were performed using MedCalc Statistical Software (MedCalc Software Ltd., Ostend, Belgium). Data were expressed as mean ± standard deviation (SD), and statistical significance was set at *p* < 0.05. The chi-square test was used to evaluate gender distribution, and the Shapiro–Wilk test assessed data normality.

Intergroup comparisons were conducted using one-way analysis of variance (ANOVA) followed by Tukey–Kramer post hoc testing. Intragroup differences between baseline (T0) and follow-up (T1) were analyzed using paired t-tests. Pearson correlation analysis was applied to evaluate relationships between clinical and biochemical parameters. All analyses met assumptions of normality and homogeneity of variance.

The full study protocol is available from the corresponding author upon reasonable request.

## Results

Patient data on periodontal parameters of patients in the three study groups, along with their initial means, standard deviations, and 95% confidence intervals (95% CI), are shown in Table 1. All periodontal parameters in SP and NP groups differed significantly from Group H at 3-month (*p* < 0.05), with no significant difference between SP and NP groups (*p* > 0.05).

**Table 1:** Changes in clinical periodontal parameters among study groups at baseline (T0) and follow-up (T1)

Group	Time														
	PI		*P* ^*^	GI		*P* ^*^	BOP %		*P* ^*^	PPD		*P* ^*^	CAL		*P* ^*^
	T_0_	T_1_		T_0_	T_1_		T_0_	T_1_		T_0_	T_1_		T_0_	T_1_	
**H**	1.04 ± 0.29^a^		-	0.67 ± 0.19^a^		-	0.05 ± 0.02^a^		-	1.81 ± 0.30^a^		-	0.00 ± 0.00^a^		-
**SP**	2.46 ± 0.46^b*^	1.67 ± 0.50^b*^	**< 0.001**	2.36 ± 0.53^b*^	1.70 ± 0.47^b*^	**< 0.001**	2.79 ± 0.74^b*^	1.38 ± 0.46^b*^	**< 0.001**	4.32 ± 0.90^b*^	3.82 ± 0.79^b*^	**< 0.001**	4.61 ± 0.95^b*^	4.14 ± 0.82^b*^	**< 0.001**
**NP**	2.48 ± 0.48^b*^	1.73 ± 0.53^b*^	**< 0.001**	2.51 ± 0.49^b*^	1.60 ± 0.48^b*^	**< 0.001**	3.24 ± 0.69^b*^	1.61 ± 0.64^b*^	**< 0.001**	4.21 ± 0.63^b*^	3.50 ± 0.86^b*^	**0.0010**	4.50 ± 0.95^b*^	3.80 ± 0.87^b*^	**0.0002**
***P*** ^***†***^	**< 0.001**	**< 0.001**		**< 0.001**	**< 0.001**		**< 0.001**	**< 0.001**		**< 0.001**	**< 0.001**		**< 0.001**	**< 0.001**	

*PI* Plaque Index, *GI* Gingival Index, *PPD* Probing Pocket Depth, *BOP* Bleeding on Probing, *CAL* Clinical Attachment Level

*H* healthy subjects, *SP* smokers with periodontitis, *NP* non-smokers with periodontitis. Different superscript letters indicate significant differences among groups at the same time point (*P* < 0.05). *Significant difference between T0 and T1 within the same group (*P* < 0.005). Data are presented as mean ± SD

Following NSPT, significant reductions were observed in all clinical parameters. PI and GI values were found to be high at T0 and significantly decreased at T1 in both SP and NP groups (*p* < 0.001). BOP values decreased significantly in both groups from 86.6 ± 16.7 (95% CI: 79.4–93.8) at T0 to 31.3 ± 8.1 (95% CI: 27.5–35.1) at T1 in the SP group, and from 77.3 ± 15.7 (95% CI: 70.6–84.0) at T0 to 32.8 ± 9 (95% CI: 28.7–36.9) at T1 in the NP group (*p* < 0.001). PPD and CAL values were also higher at T0 in both SP and NP groups, and showed a significant decrease at T1 (*p* < 0.05 for PPD, *p* < 0.001 for CAL in the SP group, *p* < 0.001 in the NP group).

**Fig. 2:**
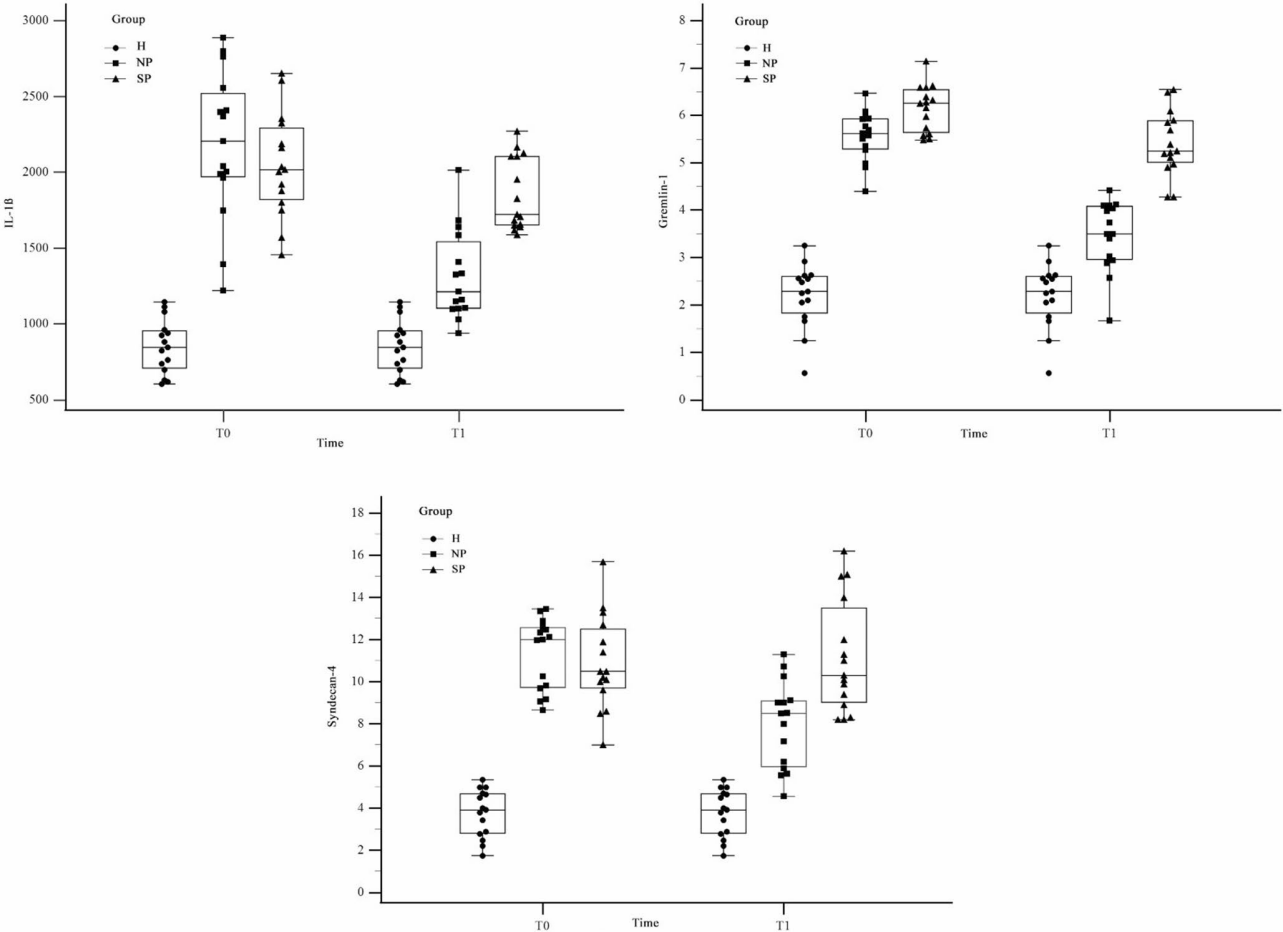
Comparison of biochemical parameters

Biochemical results are shown in Table 2; Fig. 2. In Group H, the levels of Gremlin-1, IL-1β and Syndecan-4 were measured as 2.20 ± 0.68 (95% CI: 1.92–2.48), 852 ± 178 (95% CI: 770–934) and 3.76 ± 1.12 (95% CI: 3.28–4.24) at T0, respectively.

**Table 2:** Changes in biochemical parameters among study groups at baseline (T0) and follow-up (T1)

Group	Time								
	Gremlin		*P* ^*^	IL-1β		*P* ^*^	Syndecan		*P* ^*^
	T_0_	T_1_		T_0_	T_1_		T_0_	T_1_	
**H**	2.20 ± 0.68^a^		-	852 ± 178^a^		-	3.76 ± 1.12^a^		-
**SP**	6.15 ± 0.49^b*^	5.41 ± 0.69^b*^	**0.0002**	2049 ± 343^b^	1855 ± 239^b^	**0.0817**	10.9 ± 2.24^b^	11.2 ± 2.70^b^	**0.7912**
**NP**	5.57 ± 0.52^c*^	3.47 ± 0.74^c*^	**< 0.0001**	2184 ± 490^b*^	1350 ± 297^c*^	**< 0.001**	11.3 ± 1.68^b*^	7.96 ± 2.05^c*^	**< 0.0001**
***P*** ^***†***^	**< 0.001**	**< 0.001**		**< 0.001**	**< 0.001**		**< 0.001**	**< 0.001**	

*H* healthy subjects, *SP* smokers with periodontitis, *NP* non-smokers with periodontitis. Different superscript letters indicate significant differences among groups at the same time point (P† < 0.05). Significant difference between T0 and T1 within the same group (*P* < 0.005). Data are presented as mean ± SD

In the SP group, Gremlin-1 levels decreased from 6.15 ± 0.49 (95% CI: 5.93–6.37) at T0 to 5.41 ± 0.69 (95% CI: 5.10–5.72) at T1 (*p* < 0.001), and there was no significant change in IL-1β and Syndecan-4 levels (*p* > 0.05).

In the NP group, Gremlin-1 decreased significantly from 5.57 ± 0.52 (95% CI: 5.33–5.81) at T0 to 3.47 ± 0.74 (95% CI: 3.14–3.80) at T1 (*p* < 0.0001), IL-1β decreased from 2184 ± 490 (95% CI: 1968–2400) to 1350 ± 297 (95% CI: 1217–1483) (*p* < 0.001), and Syndecan-4 decreased from 11.3 ± 1.68 (95% CI: 10.6–12.0) to 7.96 ± 2.05 (95% CI: 7.04–8.88) (*p* < 0.0001).

In comparisons between groups, T0 values of Group H were lower than the other groups for all biomarkers. At T1 NP group showed significantly lower levels than SP group, but still higher than H group (*p* < 0.001). All data are presented as mean ± standard deviation with 95% confidence intervals.

As indicated in Table 3, strong correlations were observed between periodontal parameters and biochemical parameters.

**Table 3:** Correlations between clinical periodontal parameters and biochemical biomarkers

	PI	GI	PPD	BOP %	CAL	Gremlin-1	IL-1β	Syndecan-4
PI	*	0.880	0.852	0.840	0.763	0.681	0.660	0.660
GI	0.880	*	0.939	0.921	0.827	0.733	0.738	0.729
PD	0.852	0.939	*	0.942	0.842	0.767	0.769	0.716
BOP %	0.840	0.921	0.942	*	0.789	0.793	0.787	0.725
CAL	0.763	0.827	0.842	0.789	*	0.837	0.780	0.851
Gremlin-1	0.681	0.733	0.767	0.793	0.837	*	0.847	0.832
IL-1β	0.660	0.738	0.769	0.787	0.780	0.847	*	0.749
Syndecan-4	0.660	0.729	0.716	0.725	0.851	0.832	0.749	*

*PI* Plaque Index, *GI* Gingival Index, *PPD* Probing Pocket Depth, *BOP* Bleeding on Probing, *CAL* Clinical Attachment Level

Correlation is significant at the 0.01 level (two-tailed). Correlation coefficients between 0.00–0.30 indicate weak correlation, 0.30–0.60 indicate moderate correlation, and 0.60–0.99 indicate strong correlation

## Discussion

This study investigated the levels of Gremlin-1, Syndecan-4, and IL-1β in gingival crevicular fluid (GCF) of periodontally healthy individuals and smokers and non-smokers with Stage III periodontitis before and after nonsurgical periodontal therapy (NSPT).

To our knowledge, this study represents the first simultaneous evaluation of Gremlin-1 and Syndecan-4 in GCF of smokers and non-smokers with periodontitis undergoing NSPT, addressing a critical gap in understanding smoking-related molecular healing responses.

Periodontitis is known to induce systemic inflammatory responses through the ulcerated pocket epithelium, which may further contribute to periodontal tissue destruction [19, 20]. Smoking is a major modifiable environmental risk factor that exacerbates periodontal inflammation and disease severity by impairing host immune responses [21].

Previous studies have reported inconsistent effects of smoking on clinical periodontal parameters. While some investigations observed no significant influence of smoking on plaque index (PI) before and after NSPT, [22] others reported no differences in gingival index (GI) or bleeding scores between smokers and non-smokers [23–27]. In contrast, reduced GI and bleeding parameters in smokers have also been described [28, 29]. Experimental studies demonstrated decreased gingival vascular responses in smokers during plaque accumulation [30], although post-treatment vascular density appeared comparable between smokers and non-smokers [31]. These findings suggest that the influence of smoking on gingival inflammation remains complex and multifactorial.

Similarly, studies assessing probing pocket depth and clinical attachment level after NSPT have yielded conflicting results. Some reports documented greater periodontal destruction in smokers [23, 32], whereas others observed comparable clinical outcomes between smoking and non-smoking patients [33–35]. A recent meta-analysis indicated that smokers exhibited slightly reduced attachment gain following NSPT; however, this difference was not statistically significant [36]. These discrepancies may reflect methodological differences, variations in smoking exposure, and potential behavioral influences such as the Hawthorne effect.

Regarding inflammatory biomarkers, several studies have demonstrated significant reductions in GCF IL-1β levels following NSPT [37–40]. However, the impact of smoking on IL-1β modulation remains controversial. While elevated IL-1β levels have been reported in smokers [18], other studies observed comparable inflammatory responses between smokers and non-smokers [21]. In the present study, IL-1β levels decreased significantly in non-smokers following NSPT but not in smokers, suggesting that smoking may impair inflammatory resolution and healing responses after periodontal therapy.

The nuclear factor-kappa B (NF-κB) signaling pathway plays a central role in the regulation of proinflammatory gene expression, including cytokines and adhesion molecules [41]. Gremlin-1 has been shown to contribute to inflammatory disease progression through endothelial activation [5] and to modulate NF-κB signaling in periodontal tissues [6]. In accordance with these findings, Gremlin-1 levels were significantly elevated in periodontitis groups compared with healthy controls and decreased markedly following NSPT in both smokers and non-smokers, highlighting its involvement in periodontal inflammation and therapeutic response.

Recent evidence has further demonstrated that salivary Gremlin-1 levels are significantly altered in patients with periodontitis and are associated with key clinical periodontal parameters such as plaque index, probing depth, and clinical attachment loss [42]. The authors suggested that changes in Gremlin-1 levels may reflect a compensatory regulatory mechanism aimed at modulating inflammatory activity during periodontal disease progression. While Türkmen et al. reported decreased salivary Gremlin-1 in periodontitis, we observed elevated GCF levels. This apparent discrepancy reflects compartment-specific biology: saliva represents systemic compensatory downregulation, whereas GCF reflects local inflammatory upregulation via NF-κB activation. These observations support the present findings in gingival crevicular fluid and reinforce the potential role of Gremlin-1 as a biomarker of periodontal inflammation and treatment response.

Syndecan-4 is a transmembrane proteoglycan involved in inflammatory signaling and extracellular matrix remodeling [14]. Experimental studies have shown that Syndecan-4 expression in periodontal ligament cells is dynamically regulated in response to inflammatory stimuli [43]. Although increased expression has been reported in experimental periodontitis models, its precise role in human periodontal disease remains insufficiently defined [15]. In the present study, Syndecan-4 levels were elevated in periodontitis patients and significantly decreased following NSPT only in non-smokers, suggesting a potential modulatory effect of smoking on extracellular matrix–associated inflammatory pathways.

From a biological perspective, the differential biomarker responses observed in smokers and non-smokers may reflect smoking-related alterations in inflammatory signaling and tissue healing mechanisms. Gremlin-1 has been reported to contribute to periodontal inflammation through activation of the NF-κB signaling pathway and has been suggested as a mediator of inflammatory responses in periodontal tissues [6]. Syndecan-4 is a transmembrane heparan sulfate proteoglycan involved in cell signaling, inflammation, and extracellular matrix–related interactions, and its expression in periodontal ligament cells has been shown to change dynamically in response to biological stimulation [8, 10]. IL-1β is a well-established proinflammatory cytokine that plays a central role in periodontal inflammation and bone metabolism [17]. Previous studies have also demonstrated significant reductions in IL-1β levels in gingival crevicular fluid following nonsurgical periodontal therapy [37–40]. Within this context, the absence of significant reductions in IL-1β and Syndecan-4 levels in smokers after NSPT may suggest a less favorable molecular response to therapy in this group.

Importantly, to our knowledge, this is the first clinical study to simultaneously evaluate Gremlin-1 and Syndecan-4 levels in gingival crevicular fluid of smokers and non-smokers with periodontitis before and after nonsurgical periodontal therapy. These findings provide novel insights into biomarker dynamics associated with periodontal inflammation and highlight the negative influence of smoking on molecular healing mechanisms.

Future studies should include longer longitudinal follow-up periods (e.g., 12-month designs) to better characterize long-term biomarker dynamics after periodontal therapy. In addition, evaluating dose-dependent smoking exposure may help clarify potential relationships between smoking intensity and biomarker modulation. Investigating microbiome–biomarker interactions and assessing biomarkers simultaneously in saliva and gingival crevicular fluid could further improve understanding of host–microbial mechanisms. Finally, prospective studies examining the effects of smoking cessation may help determine whether smoking-related alterations in periodontal biomarker responses are reversible.

### Limitations

This study has several limitations. The modest sample size (*n* = 15/group) and single-center design limit generalizability. Smoking status was self-reported without cotinine validation, and pack-years data were unavailable, precluding dose-response analyses. The 3-month follow-up captures short-term changes only. Microbiological assessment was not performed, limiting mechanistic insights. Effect size measures and intra-examiner reliability statistics (ICC/kappa) were not formally reported, and multivariable models adjusting for confounders were not applied. These findings should be considered preliminary pending confirmation in larger, multi-center studies with extended follow-up and comprehensive statistical modeling.

## Conclusions

Within the limitations of the present study, increased Gremlin-1 levels appear to be associated with the presence of periodontitis, and the reduction observed after nonsurgical periodontal therapy may reflect treatment-related changes in inflammatory activity. In contrast, the absence of significant changes in IL-1β and Syndecan-4 levels in smokers suggests that smoking may influence biomarker responses following periodontal therapy.

Gremlin-1 may serve as a potential biomarker for monitoring periodontal inflammatory status and treatment response. However, the biological roles and clinical relevance of IL-1β and Syndecan-4 in periodontal disease require further investigation. Overall, these biomarkers may contribute to a better understanding of periodontal inflammatory processes and could support future efforts to develop biomarker-based diagnostic or monitoring strategies in periodontal disease.

## Data Availability

AYDIN, T. (2026). Impact of Smoking on GREM1, SDC4, and IL-1β Responses to Non-Surgical Periodontal Therapy in Stage 3 Periodontitis: A Comparative Study with Periodontally Healthy Controls [Data set]. Zenodo. https://doi.org/10.5281/zenodo.18468149

## Declarations

### Clinical trial registration

Registration number is “NCT06687005”.

### Artificial intelligence

This manuscript used natural language processing tools (ChatGPT, OpenAI) solely for proofreading and improving the clarity of entirely human-generated text. No artificial intelligence was used for content generation, data analysis, or scientific interpretation.
